# Urine volume as an estimator of residual renal clearance and urinary removal of solutes in patients undergoing peritoneal dialysis

**DOI:** 10.1038/s41598-022-23093-0

**Published:** 2022-11-05

**Authors:** Joyce Pinto, Malgorzata Debowska, Rafael Gomez, Jacek Waniewski, Bengt Lindholm

**Affiliations:** 1 Nalecz Institute of Biocybernetics and Biomedical Engineering, Polish Academy of Sciences, Warsaw, Poland; 2 RTS SAS, Cali, Colombia; 3 Renal Medicine and Baxter Novum, Department of Clinical Science, Intervention and Technology, Karolinska Institutet, Stockholm, Sweden

**Keywords:** Peritoneal dialysis, Renal replacement therapy

## Abstract

In non-anuric patients undergoing peritoneal dialysis (PD), residual kidney function (RKF) is a main contributor to fluid and solute removal and an independent predictor of survival. We investigated if urine volume could be used to estimate renal clearances and removal of urea, creatinine, and phosphorus in PD patients. The observational, cross-sectional study included 93 non-anuric prevalent PD patients undergoing continuous ambulatory PD (CAPD; n = 34) or automated PD (APD; n = 59). Concentrations of urea, creatinine and phosphorus in serum and in 24-h collections of urine volume were measured to calculate weekly residual renal clearance (L/week) and removed solute mass (g/week). Median [interquartile range], 24-h urine output was 560 [330–950] mL and measured GFR (the mean of creatinine and urea clearances) was 3.24 [1.47–5.67] mL/min. For urea, creatinine and phosphorus, residual renal clearance was 20.60 [11.49–35.79], 43.02 [19.13–75.48] and 17.50 [8.34–33.58] L/week, respectively, with no significant differences between CAPD and APD. Urine volume correlated positively with removed solute masses (rho = 0.82, 0.67 and 0.74) and with weekly residual renal clearances (rho = 0.77, 0.62 and 0.72 for urea, creatinine, and phosphorus, respectively, all p < 0.001). Residual renal clearances and urinary mass removal rates for urea, creatinine, and phosphorus correlate strongly with 24-h urine volume suggesting that urine volume could serve as an estimator of typical values of residual solute removal indices in PD patients.

## Introduction

In patients on peritoneal dialysis (PD), the presence of residual kidney function (RKF) is associated with increased fluid and solute removal, improved volume status, better nutritional status, reduced erythropoietin requirements and improved survival, and RKF should therefore, whenever appropriate, be considered in the evaluation of PD patients^1,2^. While increasing the dialysis dose has failed to have an impact on the mortality of dialysis patients, RKF has consistently been a potent predictor of improved survival for both hemodialysis and PD patients^1,2^. RKF is especially important to consider when prescribing incremental PD^3–5^ and for mathematical modeling of solute kinetics during dialysis^6^.

The estimated glomerular filtration rate (eGFR) calculated by formulas based on plasma creatinine are not reliable in patients undergoing dialysis. More exact methods to measure GFR, such as inulin based, or measuring creatinine clearance following administration of cimetidine to inhibit tubular secretion, are not feasible for routine clinical evaluation^7,8^. Routine assessment of RKF in PD patients should ideally be based on measurements of the mean of 24-h urinary creatinine and urea clearances to calculate measured glomerular filtration rate, mGFR^9^. However, measurements of urea and creatinine in 24 h urine collection are often not available in clinical practice and there is a need for simpler tools, such as urine volume, to estimate RKF and to estimate the contribution of RKF to the total solute removal. Moreover, impairment of urine excretion varies from substance to substance^10–12^.

Besides the classical markers of kidney function and dialysis adequacy, urea and creatinine, the impact of residual kidney function on phosphate metabolism is of recent interest in PD^13–17^ and hemodialysis^18,19^.

Information of a potential value of urine volume as an estimate of residual small solute removal in PD patients is lacking. We explored whether correlations of urine volume with different estimations of the residual renal function for urea, creatinine, and phosphorus, could be used to assess weekly residual renal clearances and renal mass removal for investigated solutes.

## Methods

### Patients and study design

This observational, cross-sectional study included prevalent PD patients at the dialysis facilities of RTS Versalles, Cali, Colombia, who were investigated as part of their routine clinical evaluation. We included 93 non-anuric (urine output ≥ 100 mL per 24 h) patients (53.8% men, median [interquartile range] age 59 [45–67] year, body mass index 25.7 [21.3–27.7] kg/m^2^ and measured glomerular filtration rate, mGFR, 3.24 [1.47–5.67] mL/min) undergoing continuous ambulatory (CAPD, n = 34) or automated (APD, n = 59) peritoneal dialysis (Tables 1, 2). Ninety percent of patients received furosemide and the dose was 40 mg in 5%, 80 mg in 69%, 120 mg in 10%, 200 mg in 1% and 240 mg daily in 5% of the patients. Urine volume (mL/day), weekly residual renal clearance (L/week), removed solute mass (g/week), solute concentration in urine and serum for urea, creatinine and phosphorus were estimated from 24 h collections of urine and determination of solute concentrations in urine and serum. All patients received instruction on how to perform the collection of urine which—as part of the measurement of KT/V—started 6 am when urine miction was discarded and lasted with collection of all urine until 6 am the following day. Urea and creatinine were assayed by routine methods and phosphorus concentration was determined using direct UV measurement of phosphomolybdate complex.

**Table 1:** Demographic and laboratory characteristics of non-anuric patients on continuous ambulatory (CAPD), automated peritoneal dialysis (APD) and for pooled data.

	CAPD n = 34	APD n = 59	Overall n = 93
Gender, male	16 (47.1%)	34 (57.6%)	50 (53.8%)
Age, years	51 [34.3–65.8]	59 [49.5–69.0]*	59 [45.0–67.0]
Weight, kg	63.9 [54.3–71.8]	66.0 [58.0–71.0]	65.9 [57.0–71.0]
Height, m	1.6 [1.6–1.7]	1.6 [1.6–1.7]	1.6 [1.6–1.7]
Body mass index, kg⁄m^2^	25.6 [21.2–27.1]	25.7 [21.9–27.9]	25.7 [21.3–27.7]
Body surface area, m^2^	1.65 [1.53–1.79]	1.69 [1.60–1.79]	1.68 [1.55–1.79]
Total body water, L	34.1 [30.7–38.2]	35.8 [31.3–38.5]	34.4 [31–38.5]
Furosemide, mg/day^a^	80 [80–80]	80 [80–80]	80 [80–80]
Serum creatinine, mg⁄dL	8.9 [6.1–11.5]	9.3 [6.5–12.0]	9.1 [6.2–11.9]
Serum urea, mg⁄dL	45.9 [36.4–57.8]	46.2 [39.4–52.4]	46.2 [36.9–55.2]
Weekly urea KT/V	2.12 [1.91–2.58]	2.18 [1.84–2.85]	2.15 [1.87–2.71]
Serum phosphorus, mg⁄dL	5.10 [4.04–5.82]	5.36 [4.58–6.26]	5.21 [4.48–6.12]

*Statistical difference versus CAPD at p < 0.05.

^a^See “Methods” for further information on the distribution of furosemide doses.

**Table 2:** Parameters of residual renal function in patients on continuous ambulatory (CAPD), automated peritoneal dialysis (APD) and for pooled data.

	CAPD n = 34	APD n = 59	Overall n = 93
Urine volume, mL/day	580 [392.5–857.5]	560 [310–975]	560 [330–950]
Normalized urine volume, mL/day	573.62 [435.45–911.13]	549.04 [330.25–919.48]	567.06 [387.31–916.90]
mGFR, mL/min	3.26 [1.36–5.30]	3.24 [1.83–5.94]	3.24 [1.47–5.67]
**Residual renal clearance, L/week**			
Urea	21.59 [10.98–32.02]	19.67 [11.83–36.47]	20.60 [11.49–35.79]
Creatinine	41.10 [16.79–68.16]	44.25 [24.34–83.15]	43.02 [19.13–75.48]
Phosphorus	15.09 [9.08–26.99]	18.74 [8.02–34.56]	17.50 [8.34–33.58]
**Urine solute concentration, mg/dL**			
Urea	227.85 [151.85–302.50]	238.20 [190.95–284.10]	238.10 [172.10–287.30]
Creatinine	75.10 [47.69–99.96]	91.66 [65.71–124.46]	80.54 [57.16–114.38]
Phosphorus	19.82 [12.87–28.62]	23.58 [16.36–33.84]	21.30 [14.60–30.50]
**Renal mass removed, g/week**			
Urea	10.59 [4.17–13.61]	9.78 [4.80–17.78]	10.12 [4.72–17.24]
Creatinine	3.03 [1.73–4.56]	3.93 [2.13–5.95]	3.53 [1.85–5.62]
Phosphorus	0.83 [0.34–1.39]	1.08 [0.51–1.77]	1.02 [0.49–1.72]

This study extends our analyses reported previously with detailed clarification of urine volume importance as an estimate of solute removal^13,14^.

### Calculation of removed mass and residual renal clearance

Solute mass removed by the kidneys (M_renal,solute_) was evaluated from 24-h collection of urine as solute concentration in urine (C_urine,solute_) multiplied by urine volume (V_urine_): ${\text{M}}_{{{\text{renal,solute}}}}  = {\text{C}}_{{{\text{urine,solute}}}} \cdot{\text{V}}_{{{\text{urine}}}}$, where solute is urea, creatinine or phosphorus. Weekly residual renal clearance was calculated as the mass removed by the kidneys M_renal,solute_ over solute concentration in serum (C_serum,solute_) normalized using body surface area (BSA) and recalculated to one week (7 days) interval:

1 $$ {\text{Renal solute clearance }} = {\text{ 7}}\frac{{{\text{M}}_{{{\text{renal,solute}}}} }}{{{\text{C}}_{{{\text{serum,solute}}}} \cdot{\text{1week}}}}\cdot\frac{{1.73}}{{{\text{BSA}}}}\left[ {\frac{{\text{L}}}{{{\text{week}}}}} \right] $$

Residual renal function was also assessed as the measured glomerular filtration rate, mGFR, calculated as the average of urea and creatinine residual renal clearances (in mL/min).

Normalized urine volume was calculated as:

$$ {\text{Normalized urine volume }} = {\text{ Measured urine volume}} \cdot \frac{{1.73}}{{{\text{BSA}}}}\left[ {\frac{{{\text{mL}}}}{{{\text{day}}}}} \right] $$

Note that:

2 $$ {\text{Renal solute clearance }} = {\text{ }}\frac{7}{{1000}}\frac{{{\text{C}}_{{{\text{urine,solute}}}} }}{{{\text{C}}_{{{\text{serum,solute}}}} }} \cdot {\text{Normalized urine volume}}\left[ {\frac{{\text{L}}}{{{\text{week}}}}} \right] $$

### Statistical analysis

Data are expressed as median with interquartile range or as number and percentage. Chi-squared or exact Fisher test was used to compare categorical variables. Differences between continuous variable were investigated using Mann–Whitney and Kruskal–Wallis tests for two and more independent samples, respectively. Statistical dependence between variables was tested using Spearman’s correlation coefficient (rho). Observed effect was considered statistically significant at p value < 0.05; unless otherwise indicated. Statistical analyses were performed in Matlab R2021a (MathWorks, Natick, MA, USA).

### Ethical approval

All procedures performed in the study involving patients were in accordance with the Declaration of Helsinki as part of the routine clinical evaluation. Approval was granted by the RTS Ethical and Investigation Committee (September 2016). The informed consent was not required for the time of study duration, but each patient gave informed consent to perform laboratory measurements and for data management.

## Results

**Figure 1:**
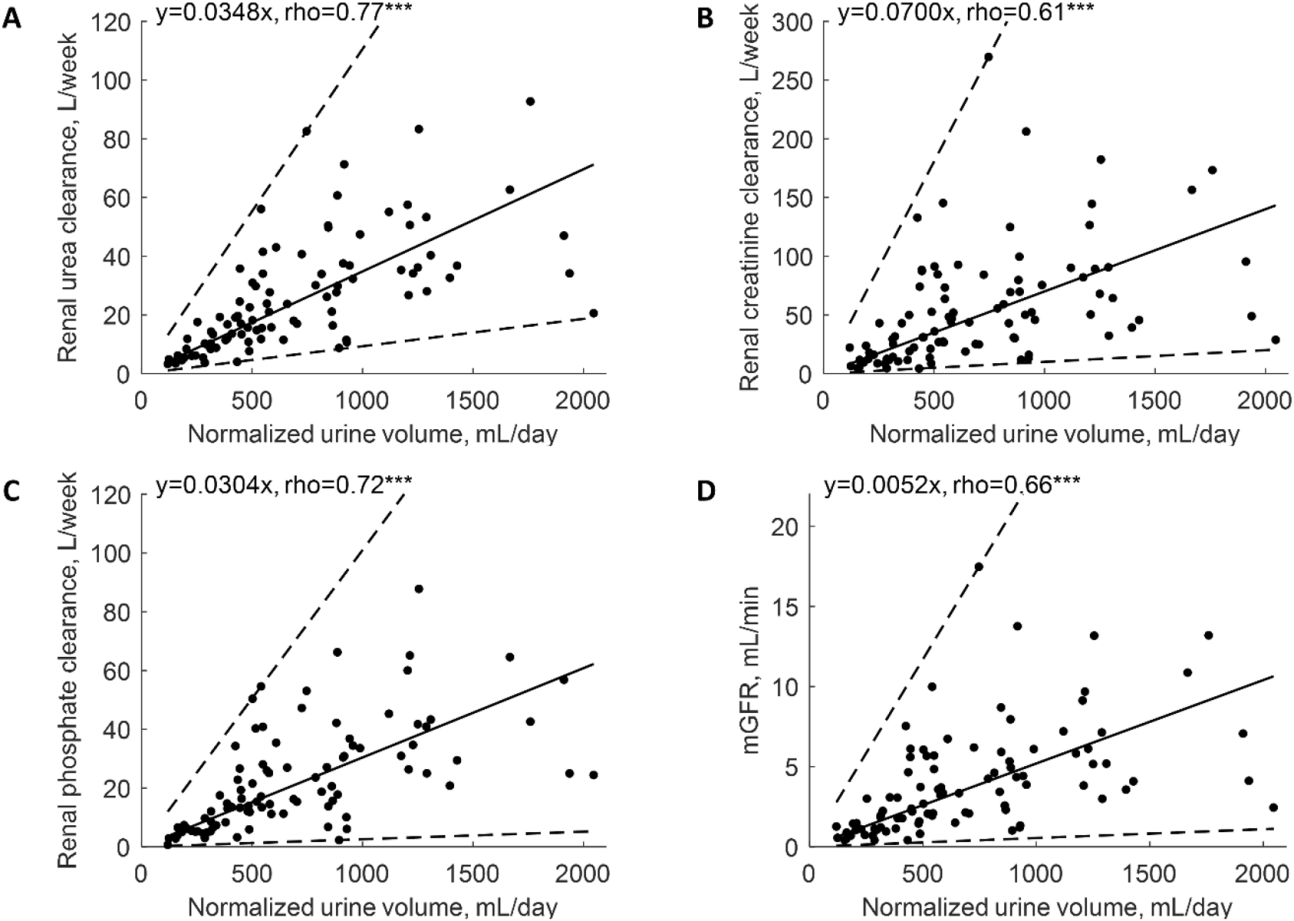
Residual renal urea clearance (panel **A**), renal creatinine clearance (panel **B**), renal phosphate clearance (panel **C**) and measured glomerular filtration rate (mGFR, panel **D**) as function of normalized urine volume. *** denote p value < 0.001. Solid line represents linear regression with equation displayed on the figure, dashed lines represent 0th and 100th percentile (minimum and maximum) of the data set.

**Figure 2:**
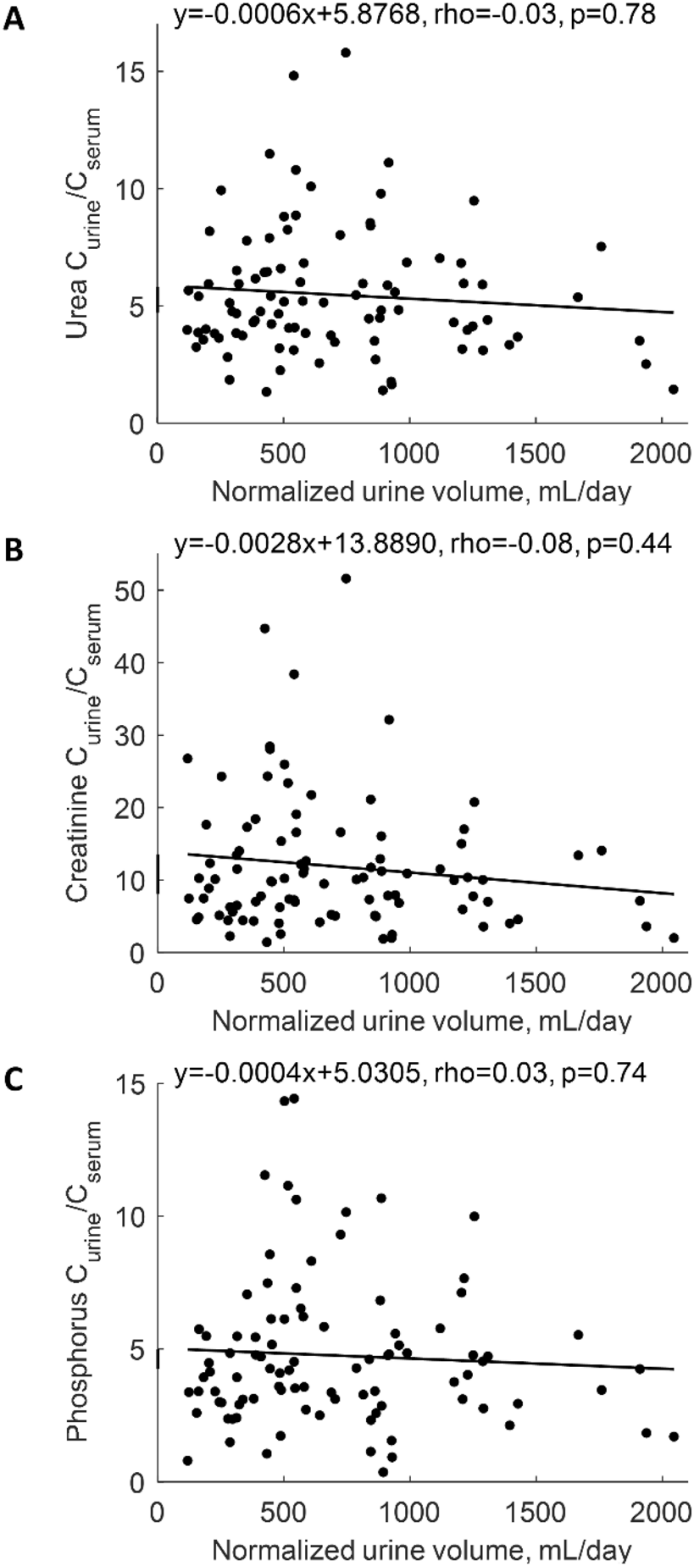
The ratio of the concentration in urine over concentration in serum (C_urine_/C_serum_) for urea (panel **A**), creatinine (panel **B**) and phosphorus (panel **C**) vs. normalized urine volume. Continuous line represents linear regression with equation displayed on the figure.

**Figure 3:**
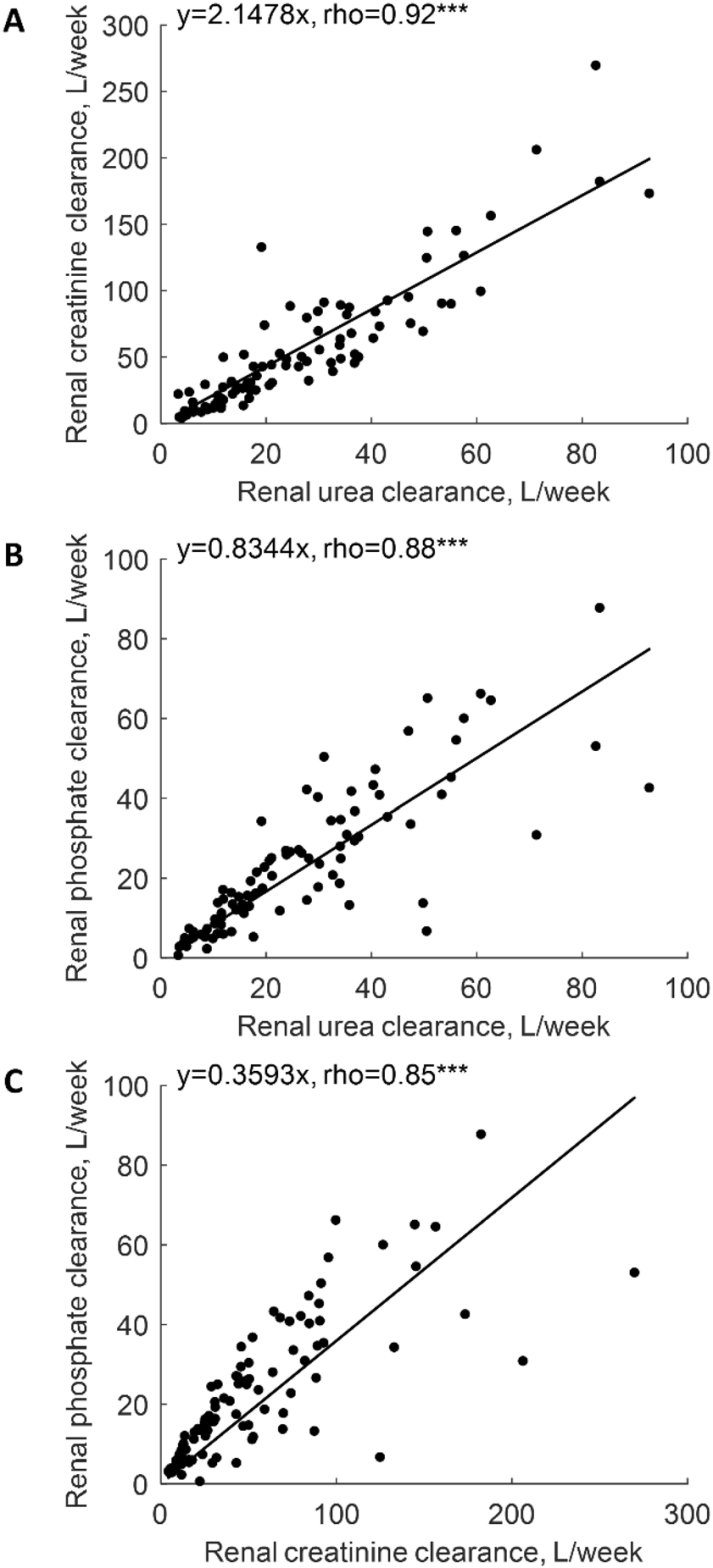
Weekly residual renal clearance of creatinine (panel **A**) and phosphate (panel **B**) vs. that of urea and renal phosphate clearance vs. renal creatinine clearance (panel **C**). *** denote p value < 0.001.

**Figure 4:**
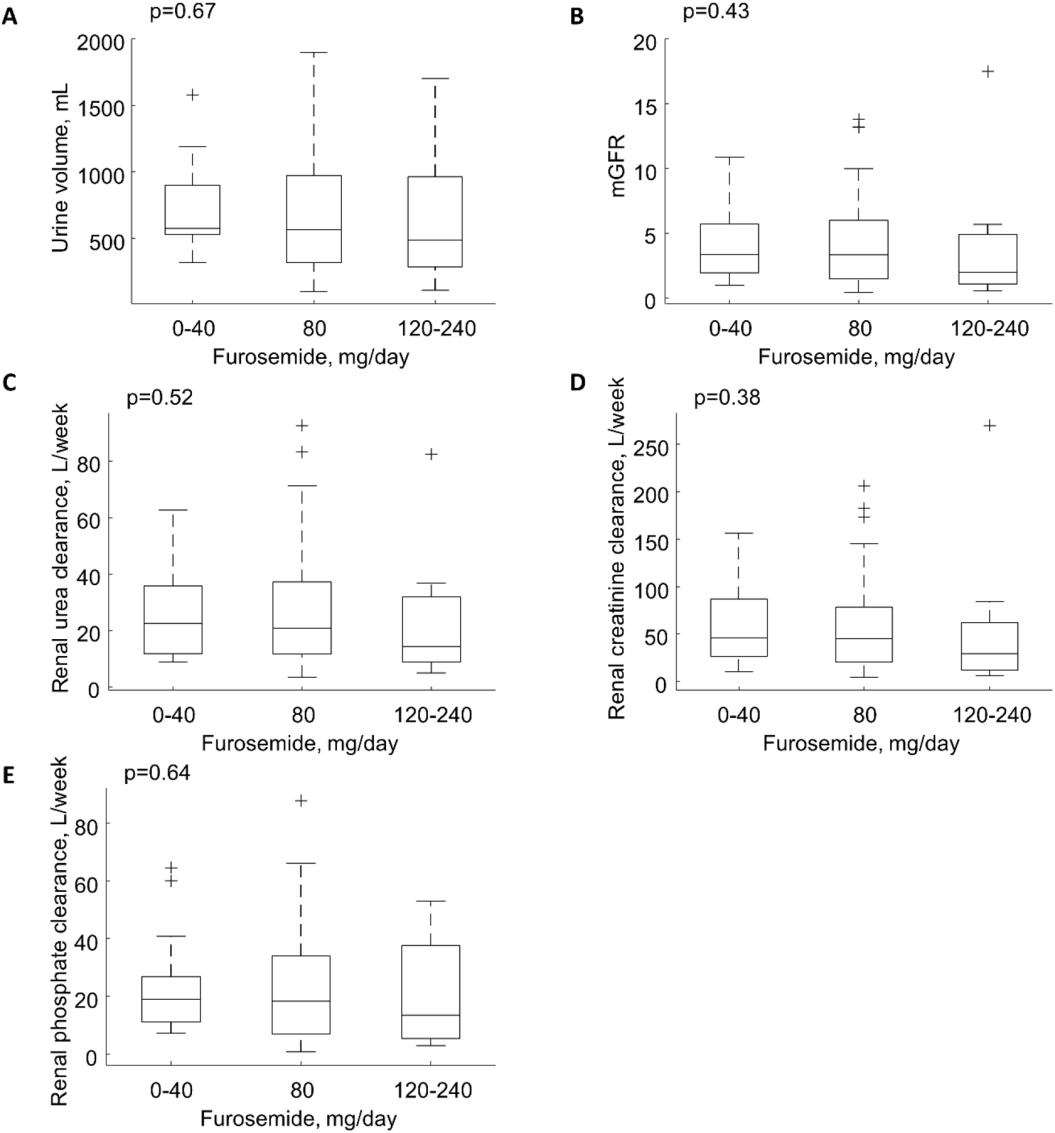
Urine volume (panel **A**), measured glomerular filtration rate (mGFR, panel **B**), renal urea (panel **C**), creatinine (panel **D**) and phosphate (panel **E**) clearance vs. daily dose of furosemide.

Median 24-h urine output was 560 [330–950] mL (Table 2). Renal mass removal for urea, creatinine and phosphorus was 10.12 [4.72–17.24], 3.53 [1.85–5.62] and 1.02 [0.49–1.72] g/week, respectively (Table 2). Serum creatinine correlated weakly and negatively with urine volume at rho = − 0.25, p < 0.05, but no such relationship was observed for urea and phosphorus (Table 3). Urine volume did not correlate with urine urea or urine phosphorus in contrast to urine creatinine that correlated (weakly) negatively at rho = − 0.28, p < 0.01 (Table 3). Urine volume correlated positively with urea, creatinine and phosphorus renal clearances at rho 0.77, 0.62 and 0.72, respectively (all p < 0.001, Table 3), and the mass of urea, creatinine and phosphorus removed by the kidney with rho = 0.82, 0.67 and 0.74, respectively (all p < 0.001, Table 3); Fig. 1A–C shows the linear regression of residual renal clearance of the solutes vs. normalized urine volume. The regression for mGFR was: mGFR [mL/min] = 0.0052 · normalized urine volume [mL/day] with rho 0.66 (p < 0.001, Table 4, Fig. 1D). The ratios of urine to serum concentration for phosphorus, creatinine and urea did not correlate with urine volume (Tables 3, 4, Fig. 2). Renal urea clearance (20.60 [11.49–35.79] L/week) correlated positively with creatinine renal clearance (43.02 [19.13–75.48] L/week), (rho = 0.92, p < 0.001), and with phosphorus renal clearance (17.50 [8.34–33.58] L/week), (rho = 0.88, p < 0.001), while renal creatinine clearance correlated positively with phosphorus renal clearance (rho = 0.85, p < 0.001), (Table 2; Fig. 3). Correlations of renal function parameters and normalized urine volume were similar as vs. urine volume, see Tables 3 and 4. No statistical difference between CAPD and APD groups were concluded unless mentioned (Tables 1, 2, 3, 4). No correlation was found between the dose of furosemide and urine volume, kidney residual clearances (Fig. 4), removed solute masses, and the urine to plasma concentration ratios for urea, creatinine, and phosphorus.

**Table 3:** Correlations between urine volume and other parameters in patients on continuous ambulatory (CAPD), automated peritoneal dialysis (APD) and for pooled data.

Spearman rho (p-value)	Urine volume		
	CAPD	APD	Overall
Body mass	0.21	0.40**	0.34***
Height	0.07	0.33*	0.23*
Body mass index	0.29^t^	0.26*	0.28**
Body surface area	0.18	0.42**	0.33**
Total body water	0.25	0.36**	0.30**
Urine urea	− 0.10	0.13	0.07
Urine creatinine	− 0.43*	− 0.21	− 0.28**
Urine phosphorus	0.13	− 0.05	0.00
Urine over serum urea	− 0.02	− 0.01	− 0.01
Urine over serum creatinine	− 0.11	− 0.02	− 0.07
Urine over serum phosphorus	0.11	0.04	0.05
Serum urea	− 0.03	0.09	0.06
Serum creatinine	− 0.31^t^	− 0.23^t^	− 0.25*
Serum phosphorus	0.16	− 0.12	− 0.03
Renal urea KT/V	0.71***	0.77***	0.76***
Renal urea clearance	0.73***	0.79***	0.77***
Renal creatinine clearance	0.57***	0.65***	0.62***
mGFR	0.63***	0.69***	0.67***
Renal phosphate clearance	0.70***	0.74***	0.72***
Renal urea removal	0.68***	0.89***	0.82***
Renal creatinine removal	0.56***	0.74***	0.67***
Renal phosphorus removal	0.71***	0.75***	0.74***

***, **, *, ^t^ denote p value < 0.001, < 0.01, < 0.05 and < 0.10, respectively.

**Table 4:** Correlations between normalized urine volume and other parameters in patients on continuous ambulatory (CAPD), automated peritoneal dialysis (APD) and for pooled data.

Spearman rho (p-value)	Normalized urine volume		
	CAPD	APD	Overall
Body mass	0.07	0.30*	0.19^t^
Height	− 0.07	0.25^t^	0.12
Body mass index	0.21	0.19	0.19^t^
Body surface area	0.03	0.30*	0.19^t^
Total body water	0.09	0.26^t^	0.18^t^
Urine urea	− 0.15	0.13	0.04
Urine creatinine	− 0.52**	− 0.23^t^	− 0.34***
Urine phosphorus	0.09	− 0.07	− 0.03
Urine over serum urea	− 0.09	0.01	− 0.03
Urine over serum creatinine	− 0.18	0.00	− 0.08
Urine over serum phosphorus	0.07	0.04	0.03
Serum urea	− 0.05	0.06	0.03
Serum creatinine	− 0.34*	− 0.26*	− 0.29**
Serum phosphorus	0.15	− 0.16	− 0.06
Renal urea KT/V	0.67***	0.80***	0.75***
Renal urea clearance	0.68***	0.81***	0.77***
Renal creatinine clearance	0.51**	0.67***	0.61***
mGFR	0.57***	0.72***	0.66***
Renal phosphate clearance	0.66***	0.75***	0.72***
Renal urea removal	0.63***	0.88***	0.78***
Renal creatinine removal	0.44**	0.72***	0.60***
Renal phosphorus removal	0.66***	0.73***	0.70***

***, **, *, ^t^ denote p value < 0.001, < 0.01, < 0.05 and < 0.10, respectively.

## Discussion

Residual kidney function (RKF) is an important contributor to fluid and solute removal and an independent predictor of survival in patients on peritoneal dialysis^1,2^ and hemodialysis^20^. While the amount of diuresis, by improving volume status, appears to have a stronger impact on survival than small solute removal in patients with kidney failure^2^, the control of plasma concentrations by urinary removal of small (sodium, potassium, phosphate) and large^21^ solutes accumulating in uremia is also of major importance for clinical outcomes. Regular measurements of urine volumes in kidney failure patients with urine production to assess kidney function and solute removal indices are of value to guide timing of dialysis initiation and adjusting dialysis prescriptions to optimize solute clearances especially in the case of incremental dialysis^1–6^.

Considering that calculations of the estimated glomerular filtration rate (eGFR) by formulas based on plasma creatinine are not reliable in patients undergoing dialysis and that measurements of solutes in 24 h urine collection are rarely available in clinical practice, we investigated if urine volume could be used to estimate residual renal clearances and urinary removal of solutes. The water and solute profiles are relatively stable in patients on PD, especially if compared to those on hemodialysis, and therefore we do not expect large variability of GFR and solute clearances in our data, as observed for interdialytic breaks between hemodialysis sessions^6,8^. In the present study, residual renal clearances and renal mass removal for urea, creatinine and phosphorus correlated strongly and positively with urine volume (Tables 3, 4, Fig. 1). This suggests that data on urine volume, if available, should be considered when investigating solute removal indices in PD patients. Among residual renal clearances for urea, creatinine, and phosphorus, two of them may be assessed based on measurements of the third one (compare Fig. 3).

The mean creatinine and urea clearance, mGFR, is a clinically useful estimator of residual GFR and as reported by Olden et al.^7^, mGFR correlated better with inulin clearance than creatinine clearance alone, and the average value of mGFR was closer to inulin clearance than creatinine clearance alone, with further improvement after addition of cimetidine. However, measurements of mGFR are rarely available in routine clinical care while measurements of urine volume are more common.

Tubular reabsorption is relatively more impaired than residual GFR in patients on dialysis with severely impaired kidney function, and, therefore, solute clearances and urine volume are more dependent on glomerular filtration in patients on hemodialysis than in the patients with normal renal function^8^. Our results confirm that urine volume is strongly associated with mGFR and solute clearances also in patients on peritoneal dialysis (Tables 3, 4, Fig. 1). More advanced methods such as urinary inulin clearance or administration of cimetidine to block tubular secretion of creatinine to obtain more exact determination of GFR provide better insight into residual kidney function but typically such studies are performed in small number of patients^2,7,8^. While urine volume can be increased by diuretics, urine volume, mGFR, residual clearances and the urine to plasma concentration ratios were not correlated to the dose of furosemide in the present study (Fig. 4). The apparent lack of any effect of furosemide on urine volume and adequacy indices may be due to the relatively low doses of furosemide used among our patients.

The relationship between residual clearance and normalized urine volume is via the ratio of solute concentrations in urine and plasma, C_urine_/C_serum_, see Eq. (2). The C_urine_/C_serum_ ratio is an important marker of kidney concentration mechanisms^22–27^. The slopes of the regression lines in Fig. 1A–C are equivalent, after rescaling, to C_urine_/C_serum_ ratios of 4.97, 10.01, and 4.34 for urea, creatinine and phosphorus, respectively. These C_urine_/C_serum_ ratios obtained from the regression lines are close to the C_urine_/C_serum_ median values 4.81, 9.98, and 4.21 for urea, creatinine and phosphorus, respectively. Note that the CKD stage 5 patients—in spite of frequently very low urine volume—retain the classical relationship with C_urine_/C_serum_ for creatinine being about twice higher than C_urine_/C_serum_ ratios for urea and phosphorus^22,23^. The data points in Fig. 1 are scattered around the regression lines but lay inside characteristics cones created by the lines associated with minimal and maximal values of C_urine_/C_serum_ ratio for each solute respectively. All these associations are possible because C_urine_/C_serum_ in our data does not correlate with (normalized) residual urine volume (Fig. 2). Note however that the few patients with high urine output (normalized urine volume > 1500 mL/day) have rather low C_urine_/C_serum_ ratios; otherwise, they would not need dialysis (Fig. 2). Our finding that the urine to plasma concentration ratios for urea, creatinine and phosphate did not depend on urine volume in patients on peritoneal dialysis is unexpected and worth further investigation.

The regression lines in Fig. 1 do not describe the individual changes of residual clearance with declining urine volume—the individual history of this relationship may be considerably more sophisticated^28^. Furthermore, because high scattering of the C_urine_/C_serum_ values (solute concentration characteristics) for the same urine volume, see Fig. 2, one cannot use these regressions for the prediction of individual residual clearances with high accuracy. However, those regression lines allow for the estimation of the typical value of residual clearance given the urine volume, and the cone-like structure of distribution of data—for the assessment of likely values of the maximum and minimum residual clearances if the urine volume is known or assumed. The predicted typical values may be compared with real individual clearances or for the comparison of the mean values for different populations of patients. One can also derive from our data what is the expected range of residual clearances for a given normalized urine volume. Similarly, the typical values and range of mGFR can be predicted as a function of normalized urine volume (Fig. 1D). Our results are presented as normalized urine volume, mGFR, and residual clearances, i.e., scaled to the body surface area (BSA); this is because GFR is typically scaled to BSA^29,30^. However, the correlations between the non-scaled parameters were similar.

Some limitations of our study should be taken into account when interpreting the results. We analyzed a relatively small group of patients using two different modalities of peritoneal dialysis, CAPD and APD. However, we did not find significant differences between CAPD and APD in basic patient characteristics (except for age) and the normalized and non-normalized urine volume, mGFR, serum and urine concentrations, residual renal clearances and removed masses of urea, creatinine and phosphorus also did not differ (Tables 1, 2). Results from correlation analysis, performed separately for CAPD and APD, were generally consistent with data derived for the whole group (Tables 3, 4). An important factor in the assessment of residual urine output is the exact timing of 24 h. Strict control of this factor may be gained during hospitalization of the patients while in our patients who collected urine at home, the timing may vary to some extent. However, if the deviation from 24 h is random, as it might be expected, its impact on the correlation parameters should not significantly compromise the results. Our study was not designed to investigate the association of urine volume and solute clearance with survival. Further studies are needed to explore if there is a cut-off value of urine volume that assures a minimal solute clearance to support incremental prescriptions.

In summary, urine volume can be considered as a marker of residual urinary removal of urea, creatinine and phosphorus. The residual clearances of these three solutes correlate well to each other; two of them may be assessed based on measurements of the third one. The solute concentration mechanisms, i.e., interrelationship between serum and urine concentrations for urea, creatinine, and phosphorus in patients on peritoneal dialysis with residual function do not depend on urine volume.

## Data Availability

The data underlying this article were provided by *RTS SAS* under permission. Data will be shared on request to the corresponding author with permission of *RTS SAS.*
